# Immune dysfunction in nucleotide excision repair disorders: an underrecognized clinical phenotype with relevance for inborn errors of immunity

**DOI:** 10.3389/fimmu.2026.1865775

**Published:** 2026-07-03

**Authors:** Raphael Rossmanith, Hermann M. Wolf, Christoph B. Geier

**Affiliations:** 1 Biomedical Sciences, University of Applied Sciences Wiener Neustadt, Tulln, Austria; 2 Doctoral School Molecular Biology and Biochemistry, Institute of Molecular Biosciences, University of Graz, Graz, Austria; 3 Faculty of Medicine, Sigmund Freud University, Vienna, Austria; 4 Center for Chronic Immunodeficiency, Medical Center – University of Freiburg, Faculty of Medicine, University of Freiburg, Freiburg, Germany; 5 University Institute of Medical Genetics, University Medicine Oldenburg, Oldenburg, Germany

**Keywords:** B cell dysfunction, cockayne syndrome (CS), hypogammaglobulinemia, inborn errors of immunity (IEI), nucleotide excision repair (NER), trichothiodystrophy (TTD), xeroderma pigmentosum (XP)

## Abstract

Nucleotide excision repair (NER) is a conserved genome maintenance pathway that removes bulky, helix-distorting DNA lesions, including ultraviolet-induced photoproducts and chemically induced adducts. By restoring DNA integrity, NER preserves transcriptional continuity and replicative fitness, thereby limiting mutagenesis, replication stress, and cell death. Inherited defects in NER genes cause rare disorders such as xeroderma pigmentosum (XP), Cockayne syndrome (CS), and trichothiodystrophy (TTD), classically associated with photosensitivity, neurodevelopmental impairment, growth failure, and, in some subtypes, marked cancer predisposition. Traditionally, clinical attention has focused on dermatologic and neurologic manifestations, whereas immune dysfunction has been regarded as peripheral or secondary. Recent clinical and immunological studies indicate that immune abnormalities may represent an underrecognized component of selected NER disorders. Reported findings include impaired vaccine responses, hypogammaglobulinemia, IgG subclass deficiency, altered B cell differentiation, CD4 lymphopenia, restricted T cell receptor repertoires, and defective dendritic cell maturation. These abnormalities are not evenly distributed across the NER spectrum and appear more prominent in selected molecular subgroups. In particular, disorders affecting transcription-associated NER functions, including TFIIH-linked conditions, may combine impaired DNA repair with reduced transcriptional capacity, thereby increasing immune-cell vulnerability during activation. By contrast, many repair-dominant XP subtypes seem to retain largely preserved baseline immune function, although clinically relevant abnormalities may become apparent during infection, vaccination, or other immune challenges. In this Mini Review, we summarize clinical evidence for immune dysfunction in NER disorders, interpret it in the context of global genome and transcription-coupled NER, and discuss implications for immunological assessment and inborn errors of immunity-oriented classification.

## 1 Introduction

Nucleotide excision repair (NER) is one of the major DNA repair pathways in eukaryotic cells and is essential for the removal of bulky, helix-distorting DNA lesions (1). These lesions include ultraviolet (UV)-induced cyclobutane pyrimidine dimers and 6–4 photoproducts (2), as well as a wide range of chemically induced DNA adducts arising from endogenous metabolism or environmental exposure (3, 4). By excising a short oligonucleotide containing the lesion and restoring DNA integrity, NER prevents mutagenesis (4), transcriptional arrest (5), replication fork collapse (6), and cell death (4). In mammalian cells, NER therefore represents a central safeguard of genome stability and cellular viability (1, 4).

Inherited defects in NER genes give rise to a spectrum of rare human disorders, most prominently xeroderma pigmentosum (XP), Cockayne syndrome (CS), and trichothiodystrophy (TTD) (7–9). These disorders are classically defined by photosensitivity and cancer predisposition in XP (7), as well as growth failure and neurodevelopmental impairment in CS and TTD (8, 9). Historically, clinical attention has focused on these hallmark features, particularly the extreme skin cancer risk in XP (7). As a consequence, other organ systems, including the immune system, received comparatively little attention. In contrast, immune dysfunction has long been viewed as peripheral or secondary in NER disorders (10). XP, in particular, has traditionally been framed as a cancer-predisposition syndrome rather than an inborn error of immunity (IEI) (7). Nevertheless, recurrent infections and increased infection-related morbidity have been reported for decades, especially in patients with TTD (9, 11). Early infectious mortality has also been described in severe TTD phenotypes (9). These observations were often attributed to failure to thrive or neurological disability rather than to intrinsic immune defects (9, 11).

Over the last decade, new clinical and immunological data have called this view into question. Advances in molecular diagnostics and standardized immunophenotyping have enabled systematic evaluation of immune function in genetically defined NER cohorts (12, 13). These studies revealed clinically relevant immune abnormalities, including impaired vaccine responses (12), hypogammaglobulinemia (12, 14), IgG subclass deficiencies (14), altered B cell compartments (12), CD4 lymphopenia (15), restricted T cell receptor repertoires (15), and defective dendritic cell maturation (16). Importantly, the available data suggest that reported immune abnormalities are not uniformly distributed across NER disorders and may be enriched in specific genetic and mechanistic subgroups, particularly those involving transcription-associated NER (TC-NER) defects (12–16). However, this interpretation remains provisional because systematic immunological assessment has been performed in only a limited number of patients and genotypes.

A central challenge in understanding immune involvement in NER disorders lies in the molecular heterogeneity of the NER pathway itself (1–4). NER is not a single uniform process but rather a coordinated network of lesion recognition, DNA unwinding, excision, and repair synthesis steps (1, 2). Moreover, several NER proteins, most notably components of the transcription factor IIH (TFIIH) complex, are multifunctional and participate not only in DNA repair but also in basal transcription (17, 18). As a consequence, pathogenic variants in NER genes may selectively impair DNA repair, disrupt transcriptional competence, or affect both processes simultaneously (17–19). This distinction may help explain why immune dysfunction appears to be more consistently recognized in TC-NER defects than in repair-dominant defects, particularly in highly proliferative immune cell populations (12–16).

## 2 Nucleotide excision repair: molecular organization and immune relevance

### 2.1 Molecular architecture of NER and functional asymmetry between global genome NER and transcription-coupled NER

NER is best understood as a multi-step repair system with several tightly coordinated components, rather than as a simple linear pathway (1–4). Two major subpathways, global genome NER (GG-NER) and transcription-coupled NER (TC-NER), share a common excision machinery but differ fundamentally in lesion recognition and biological context (5, 20). GG-NER continuously surveys the entire genome for helix-distorting lesions, regardless of transcriptional status (1, 2). Damage recognition is initiated primarily by the XPC-RAD23B complex (21), with the UV-damaged DNA-binding complex composed of DDB1 and DDB2 acting as an accessory factor that enhances recognition of UV-induced photolesions, particularly within nucleosomal DNA (22, 23). In this way, GG-NER functions as a genome-wide maintenance system that protects both transcriptionally active and inactive regions from lesion accumulation (1, 2). TC-NER, in contrast, is intrinsically coupled to transcriptional activity (5, 20). It is activated when elongating RNA polymerase II stalls at DNA lesions located within actively transcribed genes (20). Stalled polymerase complexes trigger recruitment of TC-NER-specific factors such as CSA and CSB, which coordinate transcription arrest, polymerase processing, chromatin remodeling, and repair factor assembly (20, 24, 25). Importantly, stalled RNA polymerase II complexes act as signaling platforms that amplify transcriptional stress if not efficiently resolved (26). Both GG-NER and TC-NER converge on a shared core excision reaction (1, 2). TFIIH is recruited to the lesion site and mediates local DNA unwinding through the helicase activities of XPB (encoded by ERCC3) and XPD (encoded by ERCC2) (17, 18). Lesion verification is carried out by XPA (encoded by XPA) in conjunction with replication protein A (27), followed by dual incision of the damaged strand by the structure-specific endonuclease XPF (encoded by ERCC4) complexed with ERCC-1 (encoded by ERCC1) and XPG (encoded by ERCC5) (28). The resulting single-stranded gap is then filled by DNA synthesis and sealed by ligation, restoring DNA integrity (1, 2). Despite the shared excision machinery, the biological consequences of failure in these two branches are not the same. GG-NER primarily safeguards genome integrity during DNA replication (4, 6). Failure of GG-NER allows lesions to persist and be encountered during replication, increasing replication stress and reliance on damage-tolerance pathways (5, 7). Failure of TC-NER, in contrast, leads to persistent transcriptional arrest and can trigger a self-reinforcing stress state through prolonged polymerase stalling, backtracking, and collisions with replication forks (20, 26, 29). This distinction may be particularly relevant in immune cells, which repeatedly undergo rapid transitions in transcriptional output and proliferative state during activation (30, 31).

### 2.2 Immune cell vulnerability under replicative and transcriptional stress in NER disorders

Immune activation places unusually high demands on cellular function (30). Upon antigen encounter, naïve lymphocytes exit deep quiescence and rapidly initiate DNA replication and high-output transcription (31). Within hours, thousands of genes are induced, including cytokines, signaling adaptors, metabolic regulators, and lineage-defining transcription factors (31). This transition is associated with increased replication stress and a higher likelihood of transcription-replication conflicts, particularly at long and highly transcribed genes (29, 32, 33). Whether this creates a disease-relevant vulnerability in specific NER genotypes remains incompletely established and should be interpreted in light of the limited clinical evidence.

In repair-dominant NER defects, bulky DNA lesions can persist and stall replication forks during the proliferative burst associated with immune activation (4, 6). This activates ATR-dependent DNA damage response pathways and may reduce clonal expansion or promote apoptosis of damaged precursors (34). Clinically, however, many classic XP complementation groups do not present with overt immunodeficiency (7). One possible explanation is that damage-tolerance pathways may partly compensate for these defects, or that cells carrying excessive damage may be eliminated during immune development or activation. However, this remains speculative and has not been systematically tested across XP complementation groups. However, repair-dominant NER defects may also be associated with clinically relevant immune abnormalities. Our recent patient-level reports of antibody deficiency in XPA deficiency demonstrated that clinically meaningful immune impairment can occur when immune function is examined carefully, particularly in early childhood or under conditions of repeated immune challenge such as vaccination (14). In TC-NER defects, the available data suggest that immune abnormalities may be more frequently reported and more consistently detectable than in many repair-dominant NER defects. This observation should be interpreted cautiously because the available studies are small and unevenly distributed across genotypes.

TFIIH is required not only for TC-NER but also for transcription initiation and promoter escape (17–19). Through its association with the CDK-activating kinase (CAK) module, TFIIH regulates phosphorylation of the RNA polymerase II C-terminal domain and thereby controls global transcriptional capacity (17–19). Pathogenic ERCC2 variants associated with TTD can destabilize TFIIH-CAK interactions and may impair cooperation with RNA-processing assemblies involved in resolving DNA-RNA hybrids (12, 17–19). Based on related mechanistic studies, impaired resolution of DNA-RNA hybrids could contribute to R-loop accumulation, transcriptional stress, and reduced transcriptional output, although the extent to which this mechanism operates in patient immune cells remains to be defined (35–37).

In immune cells, which undergo rapid transcriptional and proliferative remodeling during activation (31), such additional stress could plausibly impair activation-induced gene expression programs, B cell differentiation, or antibody production. At present, however, this remains an interpretative model supported by limited patient-level and functional data rather than a fully established disease mechanism (12, 14).

Transcriptomic analyses of ERCC2-deficient peripheral blood mononuclear cells support a cell-intrinsic mechanism of immune dysfunction (12). In addition to effects on adaptive immune-cell fitness, persistent DNA damage may also influence innate immune signaling. In several genome instability disorders, unresolved DNA damage, replication stress, or defective processing of damaged DNA can promote the formation of micronuclei or other cytosolic DNA species, which are sensed by cyclic GMP–AMP synthase (cGAS) and activate stimulator of interferon genes (STING)-dependent type I interferon responses (38–40). Such pathways can contribute to chronic innate immune activation, inflammatory cytokine production, and senescence-associated inflammatory programs (41). In the context of NER disorders, direct evidence for a dominant cGAS–STING-driven phenotype remains limited. Nevertheless, persistent transcription-blocking lesions, unresolved transcription–replication conflicts, or impaired recovery from RNA polymerase II stalling could, in principle, provide sources of nuclear stress that secondarily engage cytosolic DNA-sensing pathways. This mechanism may therefore represent a plausible modifier of immune dysregulation rather than an established primary driver of immunodeficiency in NER disease (39–44).

**Figure 1:**
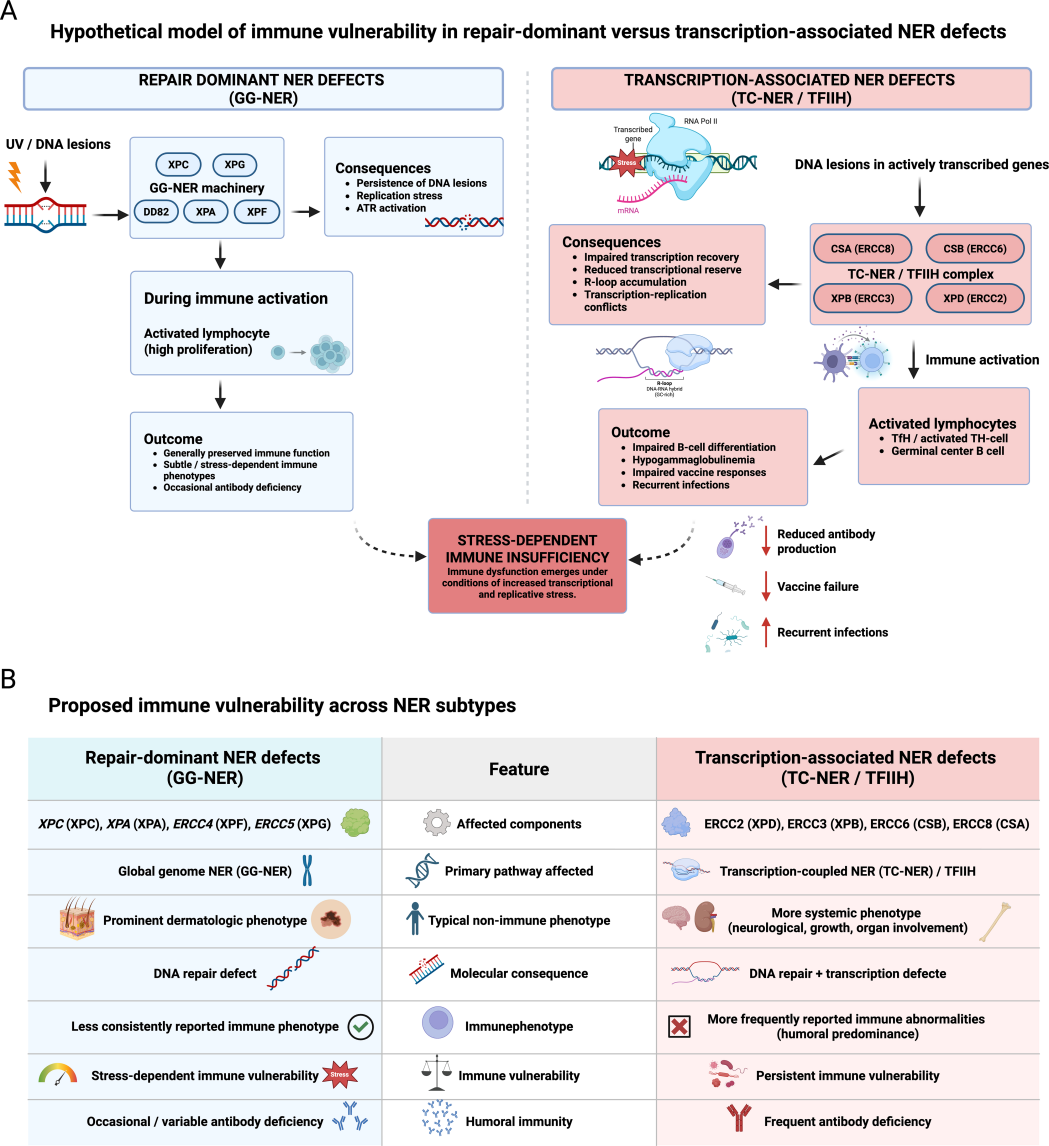
Conceptual model of immune vulnerability in nucleotide excision repair disorders. **(A)** Hypothetical framework comparing repair-dominant and transcription-associated nucleotide excision repair (NER) defects. In repair-dominant NER defects, primarily affecting global genome NER (GG-NER), persistent DNA lesions may promote replication stress and DNA damage response activation during immune-cell proliferation, while baseline immune function may often remain clinically preserved. In transcription-associated NER defects, including defects involving transcription-coupled NER (TC-NER) and TFIIH-linked functions, impaired transcription recovery, reduced transcriptional reserve, R-loop accumulation, and transcription–replication conflicts may increase vulnerability of activated lymphocytes. These stress-dependent mechanisms may contribute to impaired B-cell differentiation, reduced antibody production, defective vaccine responses, and recurrent infections. The model should be interpreted as a proposed framework rather than an established mechanism across all NER genotypes. **(B)** Proposed comparison of immune vulnerability across selected NER subtypes. Repair-dominant NER defects are typically associated with prominent dermatologic phenotypes and less consistently reported immune abnormalities, whereas transcription-associated defects, particularly TFIIH- or TC-NER-related disorders, are more often associated with systemic phenotypes and reported humoral immune abnormalities. The comparison summarizes current evidence patterns and does not imply immunological normality in genotypes that have not been systematically assessed. GG-NER, global genome nucleotide excision repair; TC-NER, transcription-coupled nucleotide excision repair; TFIIH, transcription factor IIH; XP, xeroderma pigmentosum; TTD, trichothiodystrophy; CS, Cockayne syndrome; RNA Pol II, RNA polymerase II.

These mechanisms provide a plausible framework for interpreting reported immune abnormalities in selected NER disorders. However, direct experimental evidence in patient-derived immune cells remains limited, and the relative contribution of transcriptional stress, replication-transcription conflicts, and DNA-RNA hybrid accumulation remains to be determined. A conceptual overview of the proposed framework linking NER pathway defects, transcriptional and replicative stress, innate immune sensing, and downstream immune phenotypes is provided in **Figure 1**.

### 2.3 B cell dysfunction and humoral immune phenotypes in NER disorders

Across reported NER-associated immune phenotypes, antibody deficiency and impaired vaccine responses are among the most recurrent findings (9, 12, 14). A possible explanation for these observations is that B cell responses may be sensitive to limitations in DNA repair capacity, transcriptional competence, and stress resolution during immune activation. However, this should be viewed as a mechanistic hypothesis rather than as a proven explanation for all reported humoral phenotypes. Activated B cells undergo rapid proliferation, major transcriptional reprogramming, and differentiation into antibody-secreting and memory compartments, all of which impose substantial cellular demands (45–47). Effective humoral immunity therefore depends not only on intact antigen recognition and signaling but also on the capacity to sustain activation-induced transcriptional programs and to tolerate associated replicative and transcriptional stress. Where reported, humoral abnormalities appear heterogeneous and include both broader reductions in serum immunoglobulin levels and more restricted defects, such as IgG subclass deficiency or impaired vaccine-specific antibody responses. The available data are currently insufficient to assign these patterns consistently to a specific stage of B cell activation, class-switch recombination, plasmablast differentiation, or long-lived plasma-cell maintenance.

Although nucleotide excision repair is not the canonical pathway required for double-strand break repair during the formation of the variable regions of the T- and B cell receptors which is performed during the genomic recombination of randomly used variable (V), diversity (D), and joining (J) gene segments (=V(D)J recombination) as well as class-switch recombination during B cell activation (48), B cell responses rely on the maintenance of transcriptional continuity and cellular fitness throughout activation and differentiation (29, 32). In transcription-associated NER defects, reduced transcriptional competence and impaired handling of transcription-associated stress may compromise these processes and thereby contribute to impaired antibody production, defective vaccine responses, and altered B cell differentiation (12, 14, 34–36). This interpretation is consistent with the observation that humoral immune abnormalities are among the most frequently reported immune findings in the currently available literature. However, the apparent predominance of humoral abnormalities may also reflect ascertainment bias, incomplete T cell phenotyping, and the small number of systematically studied patients (12, 14). An important unresolved question is why humoral abnormalities appear more prominent than overt T cell defects, although activated T cells also undergo extensive proliferation, metabolic remodeling, and transcriptional activation (30, 31). Several non-mutually exclusive explanations should be considered. First, T cell abnormalities may be underrecognized because routine clinical assessment often focuses on absolute lymphocyte counts and broad CD4/CD8 subsets, whereas more subtle defects in clonal expansion, exhaustion, cytokine production, Tfh differentiation, or repertoire diversity may require dedicated functional assays (15, 30, 31). Second, clinically apparent antibody deficiency may represent an integrated downstream readout of several cellular processes, including antigen presentation, CD4 T cell help, Tfh differentiation, germinal center formation, B cell proliferation, class-switch recombination, plasmablast differentiation, and plasma-cell survival (45–50). Therefore, a humoral phenotype does not necessarily imply an isolated B cell-intrinsic defect. Third, activated lymphocytes exposed to unresolved DNA damage or transcriptional stress may undergo DNA-damage-response activation, p53-dependent cell-cycle arrest, apoptosis, or senescence-like programs, which could reduce the efficiency of clonal expansion and terminal differentiation (29, 32–37). Finally, metabolic stress may further limit immune-cell fitness during activation, particularly in germinal center reactions where repeated proliferation and selection require coordinated transcriptional, metabolic, and DNA-damage-response programs (30, 31, 45–47, 50).

At present, the available evidence does not define at which stage B cell function is primarily affected, whether during early activation, germinal center responses, plasmablast differentiation, or memory formation. However, the high proliferative and transcriptional demands of activated B cells provide a plausible explanation for the predominance of humoral abnormalities in NER disorders. Even partial limitations in transcriptional capacity or cellular stress handling could, in principle, contribute to impaired humoral immunity, but this possibility requires further validation in genotype-defined patient cohorts and experimental immune-cell models.

### 2.4 Clinical evidence and genotype-associated immune phenotypes in NER disorders

The clinical evidence for immune dysfunction in NER disorders remains limited but increasingly coherent across selected genetic subgroups. Historically, literature has been dominated by dermatological, neurological, and developmental manifestations, and immune features were either incompletely characterized or interpreted as secondary consequences of severe multisystem disease (9–11). To contextualize the available clinical evidence, we summarized published reports of immune abnormalities and infectious manifestations in NER disorders. Methodological details are provided in the **Supplementary Methods**.

The most consistent currently available evidence arises from TTD, particularly ERCC2 (XPD)-associated disease, although the number of systematically studied patients remains small. Systematic reviews and cohort studies have reported frequent and sometimes severe infections, including substantial infection-associated mortality, especially in infancy and early childhood (9). More detailed immunological analyses in genetically defined ERCC2 (XPD)-deficient cohorts subsequently demonstrated hypogammaglobulinemia, impaired vaccine responses, altered B cell differentiation *in vivo*, impaired B cell receptor-mediated activation ex vivo, CD4 lymphopenia, restricted T cell receptor repertoires, and defective dendritic cell maturation (12, 13, 15, 16). Taken together, these findings support the cautious interpretation that immune dysfunction in ERCC2-associated TTD may extend beyond nonspecific infectious susceptibility and may include measurable abnormalities affecting both humoral and, to a lesser extent, cellular immune compartments.

In XP, immune involvement has historically received less attention. Earlier reports described altered interferon responses, T cell functional abnormalities, and natural killer cell dysfunction in subsets of patients (10, 11, 51). These studies were informative but often predated modern molecular classification and systematic immunophenotyping. More recently, patient-level reports have identified antibody deficiency in XPA deficiency presenting in early childhood, as well as a milder humoral phenotype in adulthood associated with ERCC4 (XPF)-deficiency (14). These reports are relevant because they show that immune dysfunction is not confined to TC-NER disorders and can also occur in more repair-dominant genotypes, although the evidence remains limited.

Available data for CS is more limited. Recurrent and severe infections, including infection-associated early mortality, have been described in severe phenotypes associated with ERCC6 (encoding CSB).

and ERCC8 (encoding CSA) defects (8, 9). However, dedicated immunophenotyping in these disorders remains sparse, and the extent to which infectious morbidity reflects intrinsic immune dysfunction, broader multisystem disease severity, or a combination of both is not yet well resolved. This distinction is important when interpreting literature, particularly in disorders with major growth, neurological, and epithelial involvement.

Across the currently available reports, immune abnormalities are therefore not uniformly distributed across the NER spectrum. Instead, they appear most consistently recognized in disorders affecting transcription-associated functions, particularly ERCC2-related TTD, while evidence for many other NER genotypes remains fragmentary. However, this pattern should not be overinterpreted. It does not establish that repair-dominant NER defects are immunologically irrelevant. More likely, it reflects small patient numbers, ascertainment bias, and the fact that systematic immunological work-up has been performed in only a minority of NER subgroups.

An additional clinically relevant observation concerns the timing of presentation. In several reported patients, recurrent infections, poor vaccine responses, or antibody deficiency were apparent in infancy or early childhood and in some cases preceded the full recognition of a syndromic DNA repair disorder (9, 12, 14). This suggests that immune manifestations may contribute to the earliest clinically detectable phenotype in selected patients and should not be regarded solely as late or secondary complications. Because the available evidence is uneven across genotypes, **Table 1** distinguishes reported immune abnormalities from infectious phenotypes and includes an evidence-level comment for each genotype. This was done to separate well-supported immune findings from infection-based observations or genotypes in which dedicated immunophenotyping remains limited, and to avoid implying immunological normality where immune involvement has not been systematically assessed. A structured overview of published immune findings across NER-associated genotypes is provided in **Table 1**.

**Table 1:** Immune and infectious phenotypes reported in selected nucleotide excision repair-associated genotypes.

Gene	Protein/molecular role	Main associated disorder(s)	Functional category	Reported immune findings	Reported infectious phenotype	Typical timing	Evidence level/comment	Key references
ERCC2 (XPD)	TFIIH helicase; nucleotide excision repair and transcription	Trichothiodystrophy; XP/TTD overlap	Transcription-associated	Hypogammaglobulinemia; impaired vaccine responses; altered B-cell differentiation; impaired B-cell activation; reduced antibody production; CD4 lymphopenia; restricted T-cell receptor repertoire; defective dendritic cell maturation	Severe and recurrent respiratory infections; bacterial infections; sepsis; infection-associated mortality	Infancy to childhood	Best-supported genotype with recurrent humoral and additional cellular abnormalities across cohorts and functional studies	(9, 12–16)
ERCC3 (XPB)	TFIIH helicase; nucleotide excision repair and transcription	Xeroderma pigmentosum; trichothiodystrophy	Transcription-associated	IgG subclass deficiency; impaired vaccine responses; impaired B-cell activation reported in isolated case-level observation	Not well defined	Adulthood in reported case	Evidence currently limited; dedicated immunophenotyping not systematically reported across ERCC3-associated disease	(14, 17–19)
ERCC6 (CSB)	Transcription-coupled repair; transcription recovery	Cockayne syndrome	Transcription-associated	Immune phenotype not systematically defined; recurrent infections reported	Severe infections and early infectious morbidity reported in severe phenotypes	Infancy/early childhood	Current evidence largely reflects infection burden rather than detailed immunophenotyping	(8, 9)
ERCC8 (CSA)	Transcription-coupled repair	Cockayne syndrome	Transcription-associated	Immune phenotype not systematically defined; early infectious morbidity reported	Recurrent or severe infections reported	Infancy/early childhood	Dedicated immunophenotyping appears limited	(8, 9)
XPA	Damage verification factor in core nucleotide excision repair	Xeroderma pigmentosum group A	Repair-dominant	Hypogammaglobulinemia; impaired vaccine responses; impaired B-cell activation; older reports also describe altered interferon responses and NK-cell abnormalities	Recurrent respiratory infections; viral infections in historical reports	Early childhood for antibody deficiency; childhood to adolescence in older reports	Evidence includes recent patient-level humoral phenotype plus older historical immune studies	(10, 14, 45)
ERCC4 (XPF)	Structure-specific endonuclease in nucleotide excision repair	Xeroderma pigmentosum group F	Repair-dominant	Antibody deficiency reported in isolated case; otherwise no systematic immune phenotype defined	Mild infectious phenotype in reported adult case	Adulthood	Evidence currently limited to isolated observation; broader immunophenotyping lacking	(14)
XPC	Global genome damage recognition	Xeroderma pigmentosum group C	Repair-dominant	No dedicated immunophenotyping identified	Infectious phenotype not systematically assessed	Not defined	No systematic immune assessment	(7)
ERCC5 (XPG)	Structure-specific endonuclease in nucleotide excision repair	Xeroderma pigmentosum group G; XP/CS overlap	Mixed/incompletely resolved	No dedicated immunophenotyping identified	Infectious phenotype not systematically assessed	Not defined	No systematic immune assessment	(7)

### 2.5 Clinical relevance and integration into inborn error of immunity frameworks

The recognition of immune dysfunction as a component of selected NER disorders has practical implications for clinical awareness and early diagnostic evaluation. Many affected children present initially with recurrent respiratory infections, prolonged viral illnesses, failure to thrive, or poor responses to routine vaccination before a syndromic diagnosis has been established (9, 14, 52, 53). In this setting, the possibility of an underlying DNA repair disorder with relevant immune involvement may not be considered early, particularly if classical dermatological or neurological features are still subtle or incomplete. Greater awareness of immune involvement may help clinicians consider NER disorders earlier in the diagnostic work-up. Clinically, a baseline immunological assessment may be informative in patients with suspected or genetically confirmed NER disorders, particularly in the presence of recurrent infections or unexpected infectious morbidity. A pragmatic initial evaluation could include serum immunoglobulins, IgG subclasses, lymphocyte subsets with focus on the B cell compartments, and assessment of vaccine-specific antibody responses (54, 55). In these disorders, immune competence may depend not only on intact recombination pathways but also, in selected genotypes, on the capacity of lymphocytes to sustain activation and differentiation programs (56–58). A normal single assessment does not exclude relevant immune dysfunction, particularly if abnormalities become apparent mainly during infection, vaccination, or early childhood. In selected patients, longitudinal follow-up, especially during early childhood or in relation to vaccination and infectious exposure, may be more informative than isolated cross-sectional testing.

These considerations also have implications for how NER disorders are positioned within the broader landscape of inborn errors of immunity (IEI). Existing IEI classification systems have historically focused on pathways directly required for immune development, antigen receptor recombination, lymphocyte signaling, or canonical effector mechanisms (59, 60). NER does not belong to the core machinery of V(D)J recombination (46), and this likely contributes to the fact that NER genes have not been prominently represented in recent IUIS-based classification frameworks (61–63). This distinguishes NER disorders from established DNA repair-associated IEIs such as ataxia-telangiectasia, Nijmegen breakage syndrome, DNA ligase IV deficiency, and Artemis deficiency. In these conditions, immune dysfunction is more directly linked to defective DNA double-strand break sensing or repair, impaired V(D)J recombination, abnormal lymphocyte development, radiosensitivity, or combined immunodeficiency (64–68). By contrast, NER is not part of the canonical machinery required for antigen receptor generation. The evidence for immune dysfunction in NER disorders is therefore weaker, more heterogeneous, and less directly connected to V(D)J recombination than in classical DNA double-strand break repair-associated IEIs. Their relevance for IEI-oriented clinical thinking lies mainly in selected genotype-dependent immune phenotypes, particularly impaired antibody responses and recurrent infections, rather than in a classical developmental recombination defect. Nevertheless, the emerging clinical literature indicates that selected NER disorders, especially those affecting transcription-associated functions, can be associated with clinically meaningful immune dysfunction.

At present, these phenotypes do not fit neatly into classical categories of severe developmental immunodeficiency. However, the available literature also argues against interpreting all reported infectious and immunological abnormalities solely as secondary complications of multisystem disease. In selected patients, reported findings such as hypogammaglobulinemia, impaired vaccine responses, altered B cell differentiation, CD4 lymphopenia, restricted T cell receptor repertoires, and defective dendritic cell maturation suggest that intrinsic immune dysfunction may contribute to the clinical phenotype (12–16). Mechanistically, this remains incompletely established but may be related to the high proliferative and transcriptional demands of immune activation (29–32, 45–48). In this regard, NER disorders may deserve broader recognition within IEI-oriented clinical thinking, especially when evaluating children with combined syndromic features and humoral immune abnormalities.

## 3 Discussion

Overall, the published literature suggests that immune dysfunction may be part of the phenotype in at least some NER disorders, although the evidence remains limited, heterogeneous, and unevenly distributed across genotypes. The available literature argues against assuming that infections and immune abnormalities in all affected patients are merely incidental or uniformly secondary to severe multisystem disease.

Rather, recurrent immune phenotypes have now been documented across multiple NER-associated conditions, with the strongest evidence emerging in genetically defined ERCC2-associated TTD and additional patient-level observations in repair-dominant genotypes such as XPA and ERCC4/XPF (9, 12–16). These findings expand the traditional clinical view of NER disorders beyond photosensitivity, neurodevelopmental disease, and cancer predisposition.

A recurring observation is that immune involvement is much better documented in some NER subtypes than in others. Instead, clinically meaningful immune dysfunction appears to be recognized most consistently in disorders affecting transcription-associated functions of the NER machinery, particularly those involving TFIIH-related components. This pattern is biologically plausible, because these defects affect both DNA repair and transcriptional capacity, a combination that could be particularly relevant in activated immune cells. However, the available data is not sufficient to establish this as a general mechanism across NER disorders. At the same time, the currently available literature does not justify a strict binary classification between transcription-associated and repair-dominant NER defects. Rather, this distinction should be understood as a useful interpretative framework, while recognizing that many genotypes remain insufficiently characterized from an immunological perspective.

The predominance of humoral abnormalities across reported cases is another notable and clinically important finding. Hypogammaglobulinemia, impaired vaccine responses, altered B cell differentiation, and reduced B cell activation recur more consistently than severe T cell developmental defects (12, 14). This pattern suggests that effective B cell responses may be especially vulnerable to the combined proliferative and transcriptional demands imposed during immune activation. However, the currently available evidence does not yet define at which stage B cell function is primarily affected, whether during early activation, differentiation, memory formation, or later antibody-secreting responses. In this context germinal center reactions depend on tightly coordinated interactions between proliferating B cells, T follicular helper (Tfh) cells, stromal signaling, and highly dynamic transcriptional programs (45–47, 49). In addition to impaired germinal center B cell fitness, transcription-associated stress may therefore also affect Tfh-cell support, plasmablast differentiation, and long-lived plasma cell maintenance (47, 49, 50). Activated lymphocytes in these compartments undergo profound metabolic reprogramming and sustained transcriptional upregulation, potentially rendering them vulnerable to replication stress, p53-dependent checkpoint activation, senescence-associated programs, and chronic stress signaling (69–71). Although the mechanism remains uncertain, the available reports indicate that humoral immune abnormalities can be clinically relevant in selected patients (12, 14). This does not exclude relevant T cell involvement. Activated T cells also undergo extensive proliferation, metabolic remodeling, and transcriptional activation (30, 31), and subtle defects in T cell expansion, cytokine production, Tfh differentiation, exhaustion, or repertoire diversity may be missed by routine immunophenotyping (15, 49). Future studies integrating functional T cell assays, metabolic profiling, and longitudinal immune phenotyping will therefore be important to better define the full immunological spectrum of transcription-associated NER disorders.

From a clinical perspective, one of the most important implications of the available literature is the timing of presentation. In several reported patients, recurrent infections, poor vaccine responses, or antibody deficiency were apparent in infancy or early childhood and in some cases preceded full recognition of a syndromic DNA repair disorder (9, 12, 14). This observation is highly relevant for practice. It argues that NER disorders should enter the differential diagnosis not only in children with photosensitivity or neurodevelopmental syndromes, but also in selected patients presenting with humoral immune abnormalities, unusual infectious morbidity, or unexplained vaccine failure, especially when these occur alongside growth abnormalities or additional syndromic features. In such settings, early immunological assessment may provide actionable information and support closer follow-up, tailored vaccination strategies, and, in selected cases, immunoglobulin replacement therapy.

These clinical observations also have broader implications for the field of IEI. NER defects are not part of the canonical machinery required for V(D)J recombination or classical immune development, and this has likely contributed to their underrepresentation in established IEI classification systems, including recent IUIS frameworks (47, 61–63). Even though, the published clinical data suggest that some NER disorders, particularly those affecting transcription-associated functions, do have relevant immune phenotypes. These conditions may not fit neatly into traditional categories of severe developmental immunodeficiency, but neither are they adequately captured as purely secondary infectious complications of multisystem disease. The field should therefore move toward broader recognition of selected NER disorders within IEI-oriented clinical and classificatory frameworks. At minimum, they warrant explicit consideration when evaluating syndromic patients with antibody deficiency or impaired vaccine responses, and as the evidence base expands, selected NER genotypes may merit more formal recognition in future IUIS classifications.

Several limitations should be acknowledged. The current evidence base is derived largely from case reports, small cohorts, historically heterogeneous case series, and limited functional studies. Many older reports predate comprehensive molecular diagnosis and standardized immunophenotyping. In addition, for several NER disorders, especially outside ERCC2-associated TTD, the distinction between intrinsic immune dysfunction and infection susceptibility secondary to broader systemic disease remains incompletely resolved. Importantly, absence of reported immune abnormalities in many NER genotypes should not be interpreted as evidence of immunological normality, but rather as a reflection of limited and uneven ascertainment.

Future work should therefore focus on systematic immunophenotyping across a broader range of NER genotypes, longitudinal follow-up beginning in early childhood, and functional studies in patient-derived immune cells. Such efforts are needed not only to refine genotype-immune phenotype correlations, but also to determine which patients carry clinically actionable immune risk. Overall, the literature suggests that some NER disorders should be viewed not only as genome maintenance disorders, but also as syndromes with relevant immunological involvement. Strengthening this recognition will be important both for patient care and for more accurate integration of these disorders into the evolving landscape of IEI.
