# Supplementary material for: Immune dysfunction in nucleotide excision repair disorders: an underrecognized clinical phenotype with relevance for inborn errors of immunity
Source: Front Immunol. 2026 Jul 3;17:1865775. doi: 10.3389/fimmu.2026.1865775 (PMC13375527; doi:10.3389/fimmu.2026.1865775)
Supplement: Supplementary file 1 [file Supplementaryfile1.docx]

**Supplementary Methods:**

This Mini Review was conducted as a narrative review informed by a structured literature search. The aim was to identify published reports describing immune abnormalities, infectious manifestations, or immunological assessment in patients with nucleotide excision repair (NER) disorders.

Targeted searches were performed in PubMed and Google Scholar during manuscript preparation and updated during revision, with the final update performed in May 2026. No lower date limit was applied, because several historically relevant reports on infectious morbidity and immune phenotypes in NER disorders predate routine molecular classification. Search terms included disease-specific, pathway-specific, and immunology-related terms, including xeroderma pigmentosum, trichothiodystrophy, Cockayne syndrome, nucleotide excision repair, DNA repair, immune deficiency, immunodeficiency, hypogammaglobulinemia, vaccine response, B cell, T cell, infection, lymphopenia, and dendritic cell. Searches were performed iteratively and were refined during manuscript preparation to capture both historical reports and recently published studies relevant to immune phenotypes in NER-associated disorders.

Original articles, case reports, case series, cohort studies, and clinically informative mechanistic studies were considered for inclusion if they reported patient-level or cohort-level immune findings, infectious manifestations, or immunological assessment in genetically defined or clinically well-characterized NER disorders. Relevant review articles were screened to identify additional primary publications through reference tracking. Reports describing recurrent or severe infections without detailed immunophenotyping were considered as supportive evidence for infectious morbidity, particularly for syndromes in which formal immunological characterization remains limited.

Studies were prioritized if they provided one or more of the following: defined immunological abnormalities, such as hypogammaglobulinemia, impaired vaccine responses, altered lymphocyte subsets, or defects in cellular immune function; clinically meaningful infectious morbidity in patients with NER disorders; genotype-resolved data enabling interpretation of immune findings in the context of specific NER genes; or mechanistic data relevant to the relationship between NER, transcriptional stress, and immune cell function.

Given the narrative scope of this Mini Review, the search was not designed as a formal systematic review and was not conducted according to PRISMA methodology. No formal meta-analysis was attempted, and risk-of-bias assessment was not performed using systematic-review appraisal tools, because the evidence base consisted predominantly of case reports, small case series, cohort studies, historical reports, and mechanistic studies of variable design.
